# Nondestructive Damage Testing of Beam Structure Based on Vibration Response Signal Analysis

**DOI:** 10.3390/ma13153301

**Published:** 2020-07-24

**Authors:** Xiaohao Li, Deyu Shi, Zihang Yu

**Affiliations:** 1 School of Mechanical Engineering and Automation, Northeastern University, Shenyang 110819, China; qaz_ln@126.com (D.S.); yu201809neu@yeah.net (Z.Y.); 2 Key Laboratory of Vibration and Control of Aero-Propulsion Systems Ministry of Education of China, Northeastern University, Shenyang 110819, China

**Keywords:** vibration signal analysis, nondestructive testing, Kalman filtering, parameter identification

## Abstract

Nondestructive damage-testing technology based on vibration signal analysis makes full use of the response characteristics of wave and energy. With the advantages of wide bandwidths of response frequency and high sensitivity, the nondestructive testing technology based on vibration signal analysis has a superiority in the application for the detection and characterization of structural defects, and has become one of the important methods for the nondestructive testing of structural material defects and damage. This paper presents a novel method of detection localization and quantitative analysis for local damage in beam structures, based on the response analysis of vibration signals. A damage-detection and -identification algorithm based on a unscented Kalman filter (UKF) was designed, which greatly reduces the computational workload in the process of damage identification over that in conventional methods. The method presented in this paper has significances to widen the application scope of the nondestructive testing method, and increase the recognition efficiency and effectiveness of this kind of method in engineering.

## 1. Introduction

With the rapid development of modern industry, nondestructive testing (NDT) technology for machinery, architecture and other structures has been rapidly developed and widely applied. However, the emergence of new materials and technologies, as well as the harsh working conditions of high temperature, high pressure and high load, have put higher requirements on the development of nondestructive testing technologies [1,2]. The effective testing of materials and structural defects is crucial to ensure the safety of materials and the reliability of structures. In the early stage of defect, crack propagation is very likely to occur in the material components under cyclic loading, which may result in the local deformation and failure of materials, introducing a huge risk of the failure of the overall system structure [3,4,5]. Therefore, it is of great practical significance to use nondestructive testing technology to monitor the quality of engineering structures in the early stage without invasive sampling.

Currently, traditional nondestructive testing technologies such as ultrasonic methods, thermal wave-imaging methods, ray methods and acoustic emission methods are widely used in mechanical engineering and the construction industry [6,7,8]. However, there are some limitations in the quantitative detection and defect expression in these methods. With nondestructive testing using vibration signal wave, its vibration response can effectively excite the vibration waves of various modes in the components being tested, such as the longitudinal wave, transverse wave and surface wave [9,10]. The reference [11] took a three-layer rack structure with six layers of storage as an example, carried out the dynamic analysis on it under horizontal and vertical loads, and completed the verification of force distribution and sag moment under test. By analyzing the interaction mechanism between the vibration wave and the structural material defects, the location and quantitative expression of the structural defects can be achieved. Compared to the traditional nondestructive testing technology, the nondestructive testing technology based on the vibration signal makes full use of the characteristics of wave and energy response. It has strong advantages in the detection and characterization of structural defects. In addition, due to its response in a wide frequency bandwidth, high sensitivity, and high spatial resolution, it has become one of the most important tools in the field of the structural defects of the material and nondestructive testing of damage [12,13,14].

In recent decades, researchers have done a lot of research on nondestructive testing technologies based on vibration signal analysis and obtained many meaningful research results. Cawley and Adams [15] found that the ratio of any two-order natural frequencies would change after the damage, which was only related to the damage location and had no relevance to the degree of damage when there was only single damage or multiple damage in the same degree in the structure, based on the assumption that the damage only caused changes in stiffness. Salawu [16] pointed out that different forms of damage might cause the same change of response frequency, especially in a situation of the damage of a symmetrical position in a symmetrical structure where the frequency index creates difficulty in distinguishing the damage. Yang et al. [17] proposed a method to add mass to solve the aforementioned issue. However, this method required the addition of known mass to the structure, which essentially changes the symmetric structure into asymmetric structure. Guan et al. [18] combined strain mode with wavelet transform and applied it to the damage identification of frame structures. Gu et al. [19] proposed a working strain mode identification technology under environmental excitation, and the corresponding damage-identification method. Pandey and Biawas [20] proposed a damage-identification method based on a modal flexibility matrix and studied the impact of damage on the flexibility matrix through numerical examples. The results showed that the damage location could be accurately determined by only using the first two order modes. However, the structure of the flexibility matrix was complex, which needs further improvement for engineering applications.

This paper presents a method for the detection of the local damage position in a structure and quantitative analysis based on vibration signal analysis. A novel algorithm of the nondestructive damage identification based on the unscented Kalman filter (UKF) was designed. This paper also expands a discussion about the disadvantages of the conventional application of the extended Kalman filter (EKF) in this field, which needs to spend high computational cost to calculate the Jacobi matrix. Compared to the EKF, the algorithm presented in this paper was able to substantially reduce the computing workload in the process of the structure damage identification, and significantly broaden the scope of application of nondestructive testing methods. The proposed method can also significantly improve the recognition efficiency and effectiveness in the engineering. At the same time, the method studied in this paper can effectively avoid: (1) the problem of low accuracy caused by the limitation of modal parameters in traditional damage-identification methods; and (2) the traditional method has a large amount of calculation work, which is not conducive to online loss identification. The effectiveness of the method was demonstrated in a computational application. The results show that the damage location identification, quantitative characterization and the anti-noise performance of UKF meets the requirements for practical engineering applications.

## 2. Design of the Nondestructive Testing Algorithms Based on Vibration Signal Analysis

### 2.1. Assumption of Nondestructive Testing Based on Vibration Signal Analysis

After damage to the structure of an equipment, the mass, stiffness and other characteristics of the equipment may all be changed. If all of these changes are considered, it is bound to increase the complexity of the research and analysis. Therefore, it is necessary to reasonably and concisely characterize the impact of damage on the structure. In engineering structures, common damages, such as cracks, have a great impact on local stiffness, while the change of local mass is usually minimal. Therefore, it can be assumed that the damage only leads to change in the local stiffness, and the change of local mass can be ignored. This assumption can be expressed as in Equation (1);

(1) $$\left\{\begin{array}{c} K_{D}\neq K_{U}\\ M_{D}=M_{U} \end{array}\right.$$

where, $K_{D}$ and $M_{D}$ are the local stiffness matrix and mass matrix after damage; $K_{U}$ and $M_{U}$ are the local stiffness matrix and the mass matrix before damage. The assumptions described as Equation (1) are basically in line with current engineering practice [21,22,23,24], and this can greatly simplify the difficulty of the research. According to Equation (1), a factor of local damage in a structure can be further defined with Equation (2):

(2) $$K_{D}=\left(1-D\right)K_{U}$$

where, D is the factor matrix of local damage, and it ranges 0∼1, where 0 means no damage and 1 means complete destruction. Formula (2) describes the reduction of local stiffness caused by the damage, which is the basis for defining and simulating damage.

Based on the above assumptions, this paper has designed a nondestructive testing algorithm as follows.

### 2.2. Design of Algorithms of the Nondestructive Testing Based on Vibration Signal Analysis

With known excitation, the motion equations of a linear structure with multiple degrees of freedom can be written as

(3) $$M\ddot{X}\left(t\right)+C\dot{X}\left(t\right)+KX\left(t\right)=Bf\left(t\right)$$

where, $X\left(t\right)$, $\dot{X}\left(t\right)$ and $\ddot{X}\left(t\right)$ are the displacement, velocity and acceleration responses of the structure, respectively; M, C and K are the mass matrix, damping matrix and stiffness matrix of the structure, respectively; $f\left(t\right)$ is an external excitation vector; B is the influence matrix of external excitation (position matrix). An augmented structural state variable is introduced as

(4) $$X={\left[X_{1}^{T},X_{2}^{T},X_{3}^{T},X_{4}^{T}\right]}^{T}$$

where, $X_{1}=x$, $X_{2}=\dot{x}$ and $X_{3}={\left[k_{1},k_{2},\cdots ,k_{m}\right]}^{T}$ which is a vector consisting of all non-zero elements in the stiffness matrix; $X_{4}={\left[c_{1},c_{2},\cdots ,c_{m}\right]}^{T}$ is a vector consisting of non-zero elements in the damping matrix. Note that the effect of local damage on mass is ignored. Assuming that the damping is Rayleigh damping, it obtains:

(5) C=αM+βK

where, α and β are the mass and stiffness damping coefficients, respectively, and $X_{4}={\left[\alpha ,\beta \right]}^{T}$. Equation (3) is rewritten as Equation (6) which is represented by augmented state variables:

(6) $$\left\{\begin{array}{c} X_{1}\\ X_{2}\\ X_{3}\\ X_{4} \end{array}\right\}=\left\{\begin{array}{c} X_{2}\\ M^{-1}\left[Bf\left(t\right)-\left[{\left(C\right)}_{X_{\text{1}}}X_{\text{3}}\text{+}{\left(K\right)}_{X_{\text{2}}}X_{\text{4}}\right]{\left[{\left(C\right)}_{X_{4}}X_{2}-{\left(K\right)}_{X_{3}}X_{1}\right]}^{T}\right]\\ K^{T}\\ C^{T} \end{array}\right\}$$

where, ${\left(C\right)}_{X_{1}}$ and ${\left(C\right)}_{X_{4}}$ is the damping matrix based on $X_{1}$ and $X_{4}$; ${\left(K\right)}_{X_{2}}$ and ${\left(K\right)}_{X_{3}}$ is the stiffness matrix based on $X_{2}$ and $X_{3}$. Since ${\left(C\right)}_{X_{1}}$, ${\left(C\right)}_{X_{4}}$, ${\left(K\right)}_{X_{2}}$ and ${\left(K\right)}_{X_{3}}$ contain state variables, Equation (6) is a nonlinear equation for augmented state variables, which can be shortened as

(7) $$\dot{X}=g\left(X,f,t\right)$$

The equation of the state is obtained by integrating Equation (7) with time as

(8) $$X_{k}=X_{k-1}+\int_{t_{k-1}}^{t_{k}}g\left(X,f,t\right)dt+w_{k}$$

where, $t_{k}$ denotes the time at which the k state is; $w_{k}$ is the process noise that here is assumed as Gauss white noise, thus the covariance matrix is constant matrix Q. Equation (8) can be realized by a dynamic direct integration method in the process of designing a nondestructive testing and analysis program. Considering the placement of acceleration sensors on the structure [25,26], the observation equation is expressed as

(9) $$Z_{k}=D\dot{X}=DM^{-1}\left\{\begin{array}{c} M{\left(X_{2}\right)}_{k}\\ Bf_{k}-{\left[{\left(C\right)}_{X_{\text{1}}}X_{\text{3}}\text{+}{\left(K\right)}_{X_{\text{2}}}X_{\text{4}}\right]}_{k}{\left[{\left(C\right)}_{X_{4}}X_{2}-{\left(K\right)}_{X_{3}}X_{1}\right]}_{k}^{\text{T}}\\ 0\\ 0 \end{array}\right\}+v_{k}$$

where, $f_{k}$ represents the excitation at the time of k state; $v_{k}$ is the measurement noise that here is assumed as Gauss white noise, thus the covariance matrix is a constant matrix R. Equation (9) is also a nonlinear equation, which can be shortened as

(10) $$Z_{k}=h\left(X_{k},f_{k}\right)+v_{k}$$

The aforementioned deduction shows that after introducing the augmented state variables into the linear structure, the corresponding state equations and measurement equations become nonlinear, thus a nonlinear filtering technology must be adopted. If the extended Kalman filter (EKF) is used, two Jacobi matrices as shown in Equations (11) and (12) need to be computed:

(11) $$A={\left.\frac{\partial g\left(X,f,t\right)}{\partial X}\right|}_{X=X_{k-1\left|k-1\right.}}$$

(12) $$H={\left.\frac{\partial h\left(X,f,t\right)}{\partial X}\right|}_{X=X_{k\left|k-1\right.}}$$

Based on Equations (6) and (9), the calculation of the Jacobi matrix involves differentiating the stiffness matrix and damping matrix. The process is closely related to the position of non-zero elements in the matrices. Once the structure changes, the position of the non-zero elements in the matrices will also change, thus Equations (11) and (12) must be deducted again. Therefore, this method of deduction lacks universality. When the expression of the stiffness matrix and damping matrix is very complex, the derivation process becomes even more complicated and prone to making large errors. If the unscented Kalman filter (UKF) is used, it just needs simple matrix operations on Equations (8) and (10) in the processing. Jacobi Matrix **A** and Matrix **H** are not required to be deduced again. The workload is significantly reduced, and the program becomes more universal, indicating that UKF has obvious advantages in damage identification. Based on this, a damage-identification algorithm of the vibration signal analysis, based on the UKF, is designed as follows:

(1) Establish augmented state variables according to Equation (4); construct the state equations according to Equations (6)–(8); and construct the observation equation according to Equation (9).
(2) Estimate the initial mean $X_{0\left|0\right.}$ and covariance matrix $P_{X,0\left|0\right.}$ of the state variables, and estimate the covariance matrix Q and R of process noise and measurement noise.
(3) Identify the parameters of structural stiffness and damping by the UKF filtering algorithm:

The main processing of the UKF filtering is to use the Unscented Transformation (UT) transform to deal with the nonlinear transfer of means and covariance in a Kalman filtering process. For the nonlinear equation of state and observation described as Equations (8) and (9), the UKF is processed as follows

(I) Set the initial parameters:
  (13) $$X_{0\left|0\right.}=E\left[X_{0\left|0\right.}\right]$$
  (14) $$P_{X,0\left|0\right.}=E\left[\left(X_{0\left|0\right.}-{\bar{X}}_{0\left|0\right.}\right){\left(X_{0\left|0\right.}-{\bar{X}}_{0\left|0\right.}\right)}^{T}\right]$$
  where, ${\bar{X}}_{0\left|0\right.}$ and $P_{X,0\left|0\right.}$ represent the mean and covariance matrix of the initial estimated state variables, respectively.
(II) Construct the set of sigma points
  According to ${\bar{X}}_{k-1\left|k-1\right.}$ and $P_{X,k-1\left|k-1\right.}$ of state k−1, the sigma point set $\chi_{k-1\left|k-1\right.}^{i}$ can be constructed according to Equation (15), where superscript i denotes the order of Point i in the set:
  (15) $$\chi_{i}=\{\begin{array}{l} \bar{x},i=0\\ \bar{x}+\sqrt{\left(n-\kappa \right)\left(n+\kappa \right)}{\left(\sqrt{P_{x}P_{x}^{\text{T}}}\right)}_{i},i=1,\cdots ,n\\ \bar{x}-\sqrt{\left(n-\kappa \right)\left(n+\kappa \right)}{\left(\sqrt{P_{x}P_{x}^{\text{T}}}\right)}_{i-n},i=n+1,\cdots ,2n \end{array}$$
(III) Predict
  By substituting the sigma point set $\chi_{k-1\left|k-1\right.}^{i}$ into the Kalman filter [27,28], the nonlinear stochastic difference Equation (16) can be obtained as
  (16) $$X_{k}=f\left(X_{k-1},u_{k-1}\right)+w_{k-1}$$
  Then, the transformed sigma point set $\chi_{k\left|k-1\right.}^{i}$ can be obtained as
  (17) $$\chi_{k\left|k-1\right.}^{i}=f\left(\chi_{k\left|k-1\right.}^{i},u_{k-1}\right)$$
  According to $\chi_{k\left|k-1\right.}^{i}$, the predicted mean of state variables is shown as Equation (18) and the covariance matrix is shown as Equation (19):
  (18) $${\bar{X}}_{k\left|k-1\right.}=\sum_{i=0}^{2n}W_{i}^{\left(m\right)}\chi_{k\left|k-1\right.}^{i}$$
  (19) $$P_{x,k|k-1}=\sum_{i=0}^{2n}\sum_{m=0}^{2n}W_{i}^{\left(m\right)}\left[\chi_{k|k-1}^{i}-X_{k|k-1}\right]{\left[\chi_{k|k-1}^{i}+{\bar{X}}_{k|k-1}\right]}^{\text{T}}+\sum_{i=0}^{2n}\left(\chi_{k|k-1}^{i}-I\right){\left(\chi_{k|k-1}^{i}+I\right)}^{\text{T}}10^{-5}I$$
  where, $W_{i}^{\left(m\right)}$ is the calculated mean weight coefficient and we set $\sum_{i=0}^{2n}\left(\chi_{k|k-1}^{i}-I\right){\left(\chi_{k|k-1}^{i}+I\right)}^{\text{T}}10^{-5}I$ as Q.
  By substituting $\chi_{k\left|k-1\right.}^{i}$ into the nonlinear observation equation, Equation (9), we obtain:
  (20) $$\gamma_{k\left|k-1\right.}^{i}=h\left(\chi_{k\left|k-1\right.}^{i}\right)$$
  The mean value of the observation variables is calculated as
  (21) $$Z_{k\left|k-1\right.}=\sum_{i=0}^{2n}W_{i}^{\left(m\right)}\gamma_{k\left|k-1\right.}^{i}$$
(IV) Correct
  Compute the covariance matrix of the observation variables:
  (22) $$P_{z,k|k-1}=\sum_{i=0}^{2n}\sum_{c=0}^{2n}W_{i}^{\left(c\right)}\left[\gamma_{k|k-1}^{i}-Z_{k|k-1}\right]{\left[\gamma_{k|k-1}^{i}+{\bar{Z}}_{k|k-1}\right]}^{\text{T}}+\sum_{i=0}^{2n}\left(\gamma_{k|k-1}^{i}-I\right){\left(\gamma_{k|k-1}^{i}+I\right)}^{\text{T}}I$$
  where, $W_{i}^{\left(c\right)}$ is the variance weight coefficient and we set $\sum_{i=0}^{2n}\left(\gamma_{k|k-1}^{i}-I\right){\left(\gamma_{k|k-1}^{i}+I\right)}^{\text{T}}I$ as **R**.
  Compute the covariance matrix between the state variables and observation vectors;
  (23) $$P_{k|k-1}=\sum_{i=0}^{2n}\sum_{c=0}^{2n}W_{i}^{\left(c\right)}\left[\gamma_{k|k-1}^{i}+Z_{k|k-1}\right]{\left[{\bar{X}}_{k|k-1}-{\bar{Z}}_{k|k-1}\right]}^{\text{T}}{\left[\chi_{k|k-1}^{i}-{\bar{X}}_{k|k-1}\right]}^{\text{T}}$$
  Then, the Kalman gain matrix can be obtain by
  (24) $$K_{k}=P_{z,k\left|k-1\right.}P_{z,k\left|k-1\right.}^{-1}$$
  Update the mean of state variables:
  (25) $$X_{k\left|k\right.}=X_{k\left|k-1\right.}+K_{k}\left(Z_{k}-Z_{k\left|k-1\right.}\right)$$
  Update the covariance matrix of the state variables:
  (26) $$P_{X,k\left|k\right.}=P_{X,k\left|k-1\right.}-K_{k}P_{z,k\left|k-1\right.}K_{k}^{T}$$
  From the steps mentioned above, it only needs to calculate the state equation and observation equation in the UKF filtering process, and does not need to calculate the Jacobi matrices. This method has obvious advantages in terms of the ease of use and flexibility when the state equation is complex and non-differentiable.
(V) Judge structural damage status based on the identification results.

**Figure 1:**
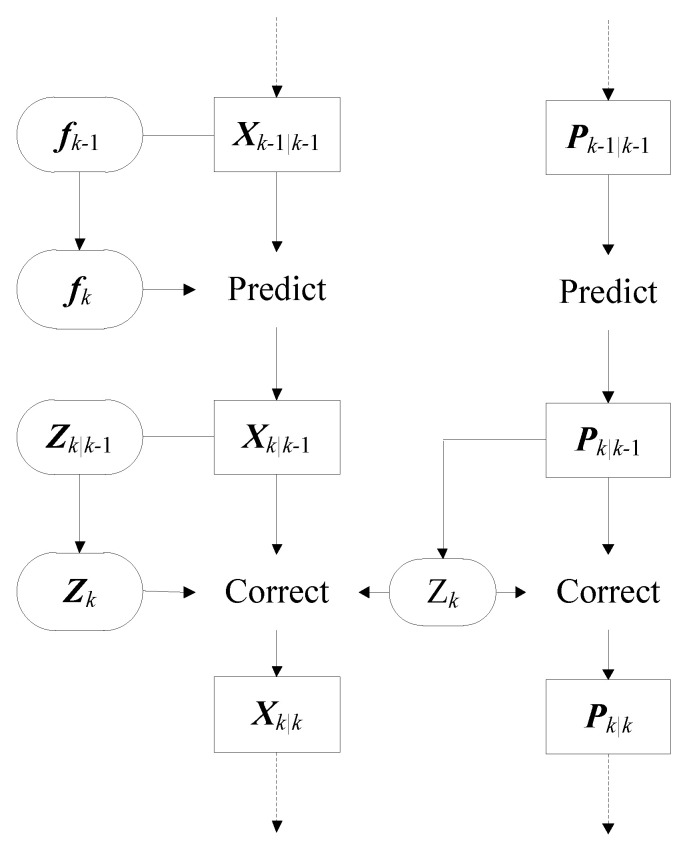
Flow chart of the unscented Kalman filter (UKF) algorithm under known excitation.

The flow chart of the UKF algorithm is shown in Figure 1.

## 3. Application Example Analysis

### 3.1. Experimental Setting

**Figure 2:**
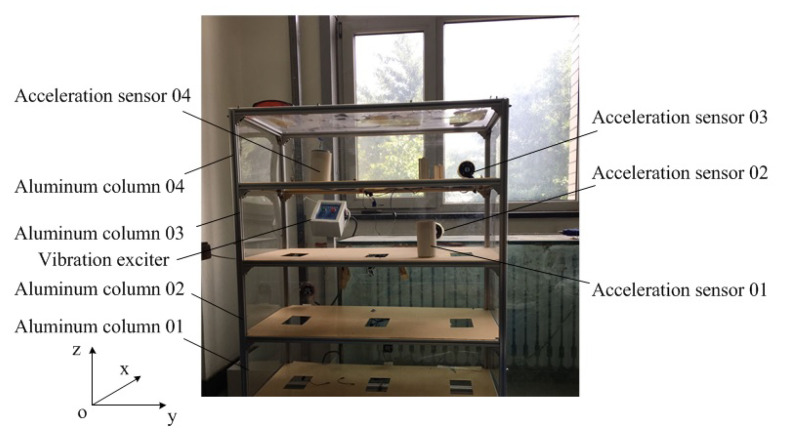
Frame structure and the sensor arrangement.

As shown in Figure 2, the four-layer frame model is composed of plexiglass plates and aluminum columns, which are connected by bolts (due to the universality of the application of a frame beam in practical production, the frame structure is taken as an example. The non-destructive testing algorithm is used to evaluate and monitor the strength, stiffness, damping and other technical parameters of the frame beam, which is of great practical significance to eliminate potential safety hazards and ensure the safety of the frame structure [29]). The structure sits on orbits and is only allowed to move in the x direction. Each layer of the structure consists of four aluminum columns (25 × 25 × 1 mm) connected with two up-and-down plexiglass plates (900 × 450 × 2.5 mm), respectively, forming a four-degree-of-freedom system.

**Figure 3:**
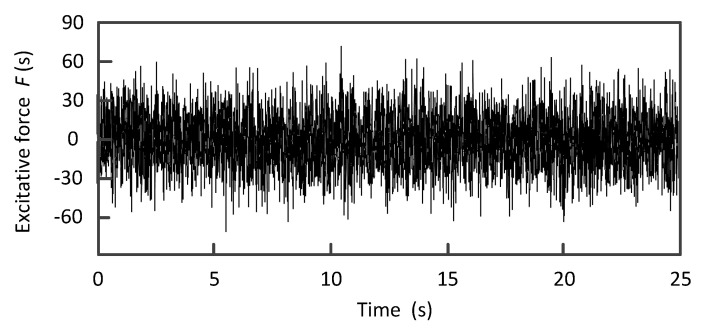
Measured random excitation signal.

In Figure 2, an electromagnetic exciter (SA-JZ-50, Wuxi Shiao Technology Co., Ltd, Wuxi, China) is used to apply the lateral excitation to the bottom plate along the center line of the structure. The structure and the exciters are installed together on the base plate. A force sensor (BK-2Y, China Academy of Aerospace Aerodynamics, Beijing, China) (sensitivity 2.23 mV/N) is installed between the rod of the exciter and the structure to measure the excitation force. Four acceleration sensors (AD100T, Qinhuangdao Xinhua Technology Co., Ltd, Qinhuangdao, China) (sensitivity 100 mV/g) are installed in the positions shown in Figure 2 to measure the acceleration response of each layer. The excitation signal is the random excitation with a bandwidth of 5~50 Hz and the excitation level is 2.6 V as shown in Figure 3.

The sampling frequency and sampling time of the experimental test system were 160 Hz and 25.6 s respectively, and 4096 points were sampled. The experiments were tested on 18 different working conditions, including increasing the mass of a certain layer, reducing the stiffness of a certain column, and introducing nonlinear damage into a buffer gap. This paper only analyzed the identification effectiveness of the algorithm on the stiffness reduction of Column 2 in Figure 2. We only selected four representative working conditions for research in this paper, as shown in Table 1.

### 3.2. Nondestructive Testing Based on Vibration Signal

**Figure 4:**
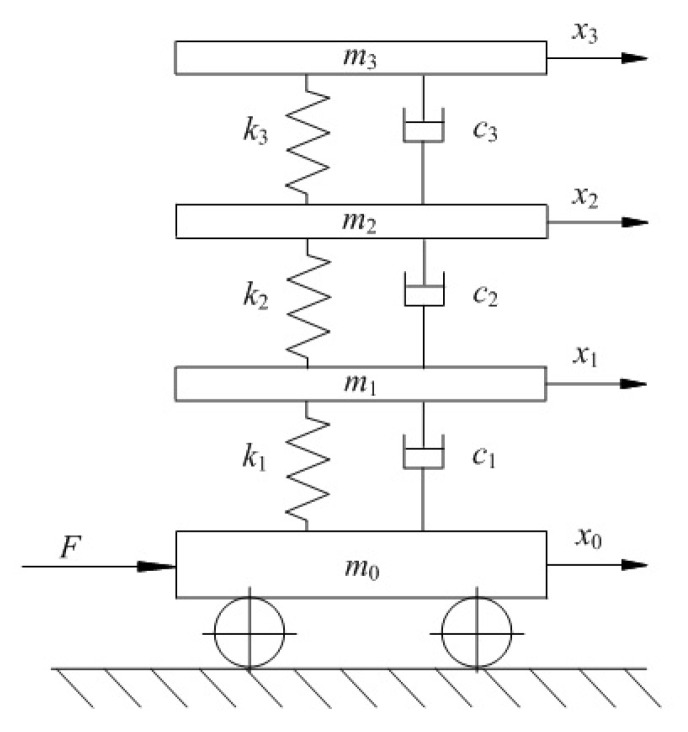
Mathematic dynamical model of the frame structure.

In order to validate the damage identification of the algorithm proposed in this paper, a numerical model of the frame structure in Figure 2 was established as shown in Figure 4, in which the friction between the bottom plate and the track is neglected.

The motion equation of the numerical model in Figure 4 is established as follows:

(27) $$M\ddot{x}+C\dot{x}+Kx=f\left(t\right)$$

where, M, C and K are the mass, damping and stiffness matrices, respectively; $f\left(t\right)$ is the input excitation vector; $x={\left[x_{0},x_{1},x_{2},x_{3}\right]}^{T}$ is the displacement of each layer, and the 0th floor represents the bottom plate; $\dot{x}$ and $\ddot{x}$ denote the speed and acceleration, respectively. The expressions of M and K are as follows:

(28) $$M=\left[\begin{array}{cccc} m_{0} & 0 & 0 & 0\\ 0 & m_{1} & 0 & 0\\ 0 & 0 & m_{2} & 0\\ 0 & 0 & 0 & m_{3} \end{array}\right],\;K=\left[\begin{array}{cccc} k_{1} & -k_{1} & & \\ -k_{1} & k_{1}+k_{2} & -k_{2} & \\ & -k_{2} & k_{1}+k_{2} & -k_{3}\\ & & -k_{3} & k_{3} \end{array}\right]$$

where, $m_{0}∼m_{3}$ denotes the mass of each layer; $k_{0}∼k_{3}$ denotes the interlayer stiffness. Damping matrix C can be assumed to be the Rayleigh damping matrix, as shown in Equation (5).

The augmented state variables as shown in Equation (4) are established by selecting the relevant displacement, velocity, story stiffness and Rayleigh damping coefficient.

For the numerical model of Figure 4, the initial values of the parameters are set according to Figure 2 as follows: (1) it is considered that the mass matrix M remains unchanged and the density of aluminum is 2700 ${\mathrm{kg}/m}^{3}$ in the processing; thus, $m_{0}=m_{1}=m_{2}=m_{3}=6.7\;\mathrm{kg}$ is calculated according to the structure size; (2) The modulus of the elasticity of aluminum is set 70 Gpa so that $k_{0}=k_{1}=k_{2}=k_{3}=4.2\times 10^{5}\;N/m$; (3) By analyzing the excitation response test of the beam element shown in Figure 2, it can be found that the measured mode damping ratio usually has less influence than the inertia and stiffness of the structure. We can determine the Rayleigh damping coefficient by using the orthogonality experiment between the damping matrix and the vibration response mode shape of the structure. Rayleigh damping coefficients are estimated as $\alpha =3.12\times 10^{-3}$, $\beta =1.77\times 10^{-4}$; (4) $Q=1.2\times 10^{-7}I$, R=1.15I, and these will be slightly and appropriately adjusted in the calculation. (5) Take the initial displacement and velocity as 0, so that $X_{0\left|0\right.}={\left[0,0,0,0,0,0,0,0,4.2\times 10^{5},4.2\times 10^{5},4.2\times 10^{5},3.12\times 10^{-3},1.77\times 10^{-4}\right]}^{T}$. By applying the magnitude balance technology, we can set $\kappa_{\mathrm{amp}}=4.2\times 10^{5}$, $\alpha_{\mathrm{amp}}=3.12\times 10^{-3}$, $\beta_{\mathrm{amp}}=1.77\times 10^{-4}$ and adjust $X_{0\left|0\right.}$ as ${\left[0,0,0,0,0,0,0,0,1,1,1,1,1\right]}^{T}$; (6) $P_{X,0\left|0\right.}$ is set as the identity matrix I.

By substituting the above parameters into the structural state equations and the observation equations that are expressed as Equations (6)~(10), the results of the NDT analysis can be obtained as described in the following section.

### 3.3. Analysis of the Nondestructive Testing Results

#### 3.3.1. Working Condition without Damage

**Figure 5:**
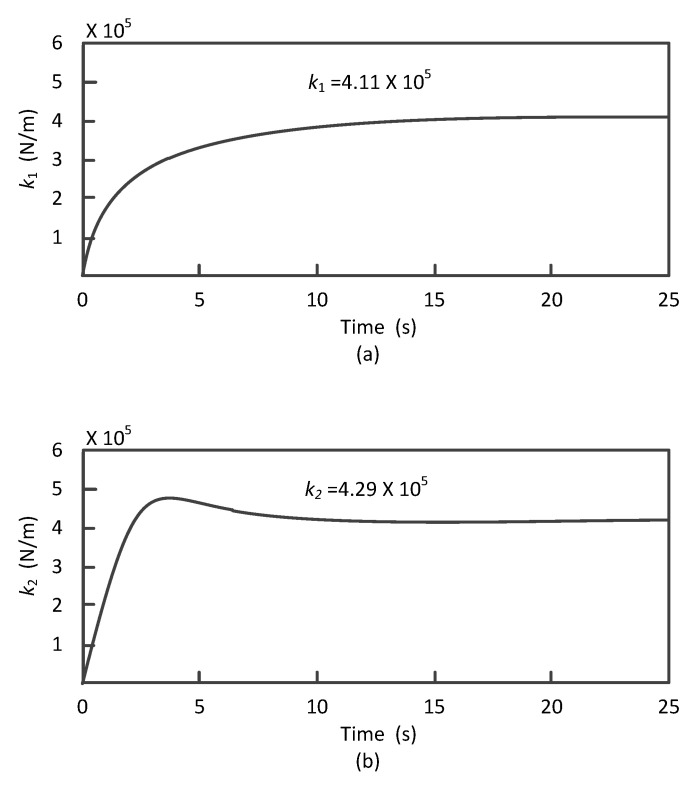

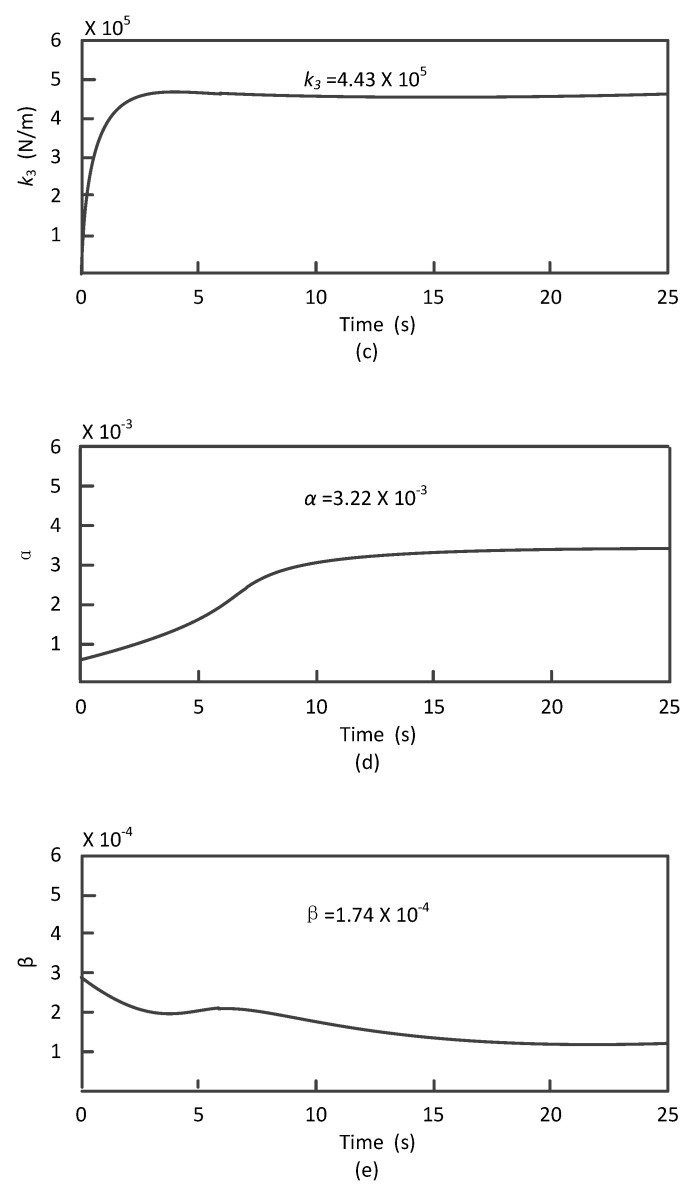
Identification results of the system parameters under the nondestructive working conditions: (**a**) recognition results of $k_{1}$, (**b**) recognition results of $k_{2}$, (**c**) recognition results of $k_{3}$, (**d**) recognition results of α and (**e**) recognition results of β.

Under the conditions without damage, $k_{1}∼k_{3}$, α and β are obtained as shown in Figure 5 when the proposed recognition algorithm is used in the situation. 

It can be seen from Figure 5 that structural damage can be identified based on the algorithm in the paper. The computation converges very quickly so that, $k_{1}∼k_{3}$ reaches the steady values within 14.5 s. Moreover, α and β converge to steady values within the 20 s. The differences between the recognition results and the initial values show that the initial estimation of the structure parameters is not accurate. However, by using the UKF it can give a more accurate structural parameter identification even under the condition with observation noises.

#### 3.3.2. Conditions of Damage on Column 2

**Figure 6:**
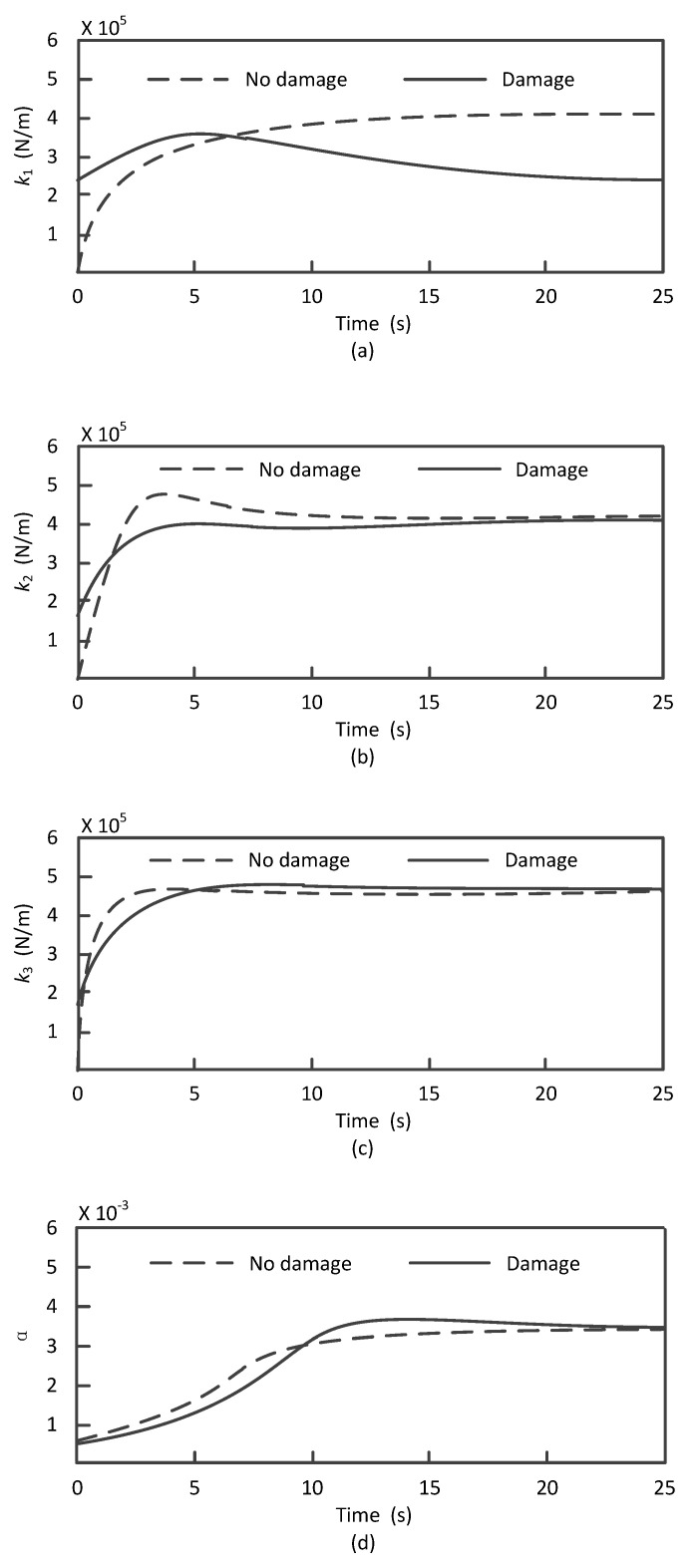

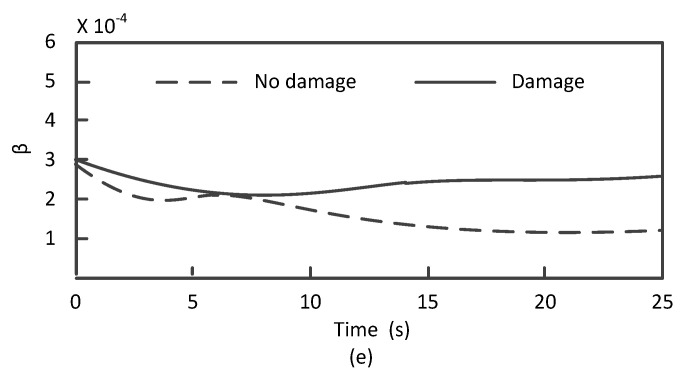
Identification of the frame’s structural parameters in the case of damage on Column 2: (**a**) recognition results of $k_{1}$, (**b**) recognition results of $k_{2}$, (**c**) recognition results of $k_{3}$, (**d**) recognition results of α and (**e**) recognition results of β.

When column 2 is damaged, the proposed algorithm is also used to identify $k_{1}∼k_{3}$, α and β. The recognition results are compared with that in Figure 5, as shown in Figure 6.

It can be seen from Figure 6 that: (1) among the stiffness parameters, only $k_{1}$ decreased considerably compared with that in the nondestructive condition. It was about $4.11\times 10^{5}\;N/m$ without damage, while about $2.53\times 10^{5}\;N/m$ after the damage, with a decrease rate of 38.4%. According to Table 1, when the stiffness of Column 2 decreases by 64.9% under Condition 2, the theoretical reduction of $k_{1}$ between the first layer and second layer should be 47%. There is a small difference between the identification by UKF and theoretical values, and this does not affect the location of damage. (2) The mass damping coefficient α does not have significant change, but the stiffness damping coefficient β changes significantly after the damage. This phenomenon was caused by the stiffness change of the frame structure which is reasonable. In conclusion, the damage-identification method based on UKF has achieved good performance, which can not only locate the damage position effectively, but also give an accurate damage degree estimation.

## 4. Conclusions

This paper presents a vibration signal analysis method based on UKF. A simulation was conducted to analyze the interaction process between the vibration and the frame structure defects. A nondestructive damage-detection algorithm based on UKF was designed in this paper. Taking the beam structure unit widely used in production as an example, the damage-identification effect of the UKF method under known excitation and unknown excitation was investigated, and the damage location and quantitative evaluation of the damage degree of the frame structure using the proposed algorithm were realized. The results show that the research method in this paper has obvious advantages in damage location and quantitative performance. Compared with the traditional Kalman filter method, the biggest advantage of the UKF method is that it does not need to calculate the Jacobi matrix, and the calculation amount is equivalent to that of EKF, but its accuracy is obviously higher than that of EKF. Thus, the calculation workload in the process of the damage identification of the frame structure is greatly reduced and the application is much simpler. In the field of structural material damage identification, the method shows a better universality. The proposed method also significantly increases the efficiency and effectiveness, as well as the damage location accuracy of damage identification in current engineering applications. The analysis of the response signal in the structure under additional noise shows that the nondestructive testing method presented in this paper can still give reliable damage-identification results under the condition of interference, showing a characteristic of strong anti-interference and high robustness. In the follow-up study, we will comprehensively analyze the material and beam structure characteristics, and use the finite element method to study the deformation, principal stress, tensile stress and shear stress of the beam element, so as to improve the practical application effect of the identification algorithm.

## Figures and Tables

**Table 1:** Physical experiment condition of the frame structure.

Working Condition Serial Number	Damage Condition
1	Nondestructive
2	The stiffness of No. 2 aluminum column decreases by 64.9%
3	The stiffness of No. 3 aluminum column decreases by 64.9%
4	The stiffness of No. 4 aluminum column decreases by 64.9%
...	...

## References

[1] Yam L.H., Li Y.Y., Wong W.O. (2002). Sensitivity studies of parameters for damage detection of plate-like structures using static and dynamic approaches. Eng. Struct..

[2] Giridhara G., Rathod V.T., Naik S., Roy Mahapatra D., Gopalakrishnan S. (2010). Rapid localization of damage using a circular sensor array and Lamb wave based triangulation. Mech. Syst. Signal Process..

[3] Wang L., Yang Z.C., Tan G.H. (2008). Improved Structural damage detection method based on natural frequency vector. J. Mech. Strength..

[4] Nie Z.H., Hao H., Ma H.W. (2013). Structural damage detection based on the reconstructed phase space for reinforced concrete slab: Experimental study. J. Sound Vib..

[5] Liu W.G., Yan L., Guo L.Q. (2016). Damage classification of elastic thin plate based on modal strain energy method. Noise Vib. Control.

[6] Aydin K., Kisi O. (2014). Damage detection in Timoshenko beam structures by multilayer perceptron and radial basis function networks. Neural Comput. Appl..

[7] Jiang J.T., Yu H.L. (2010). Study on identification of offshore platform component damage based on strain modal difference. J. Catastr..

[8] Zhang C., Cheng L., Xu H., Qiu J.H. (2016). Structural damage detection based on virtual element boundary measurement. J. Sound Vib..

[9] Satpal S.B., Guha A., Banerjee S. (2016). Damage identification in aluminum beams using support vector machine: Numerical and experimental studies. Struct. Control Health Monit..

[10] Zhao Y.X., Xu Y.G., Gao L.X. (2010). For fault pattern recognition of rolling bearing acoustic emission technique based on harmonic wavelet packet and BP neural network. J. Vib. Shock.

[11] Kostrzewski M. Loads Analysing In Pallet Racks Storage Elevation. Proceedings of the Carpathian Logistics Congress Proceedings (Reviewed Version).

[12] Friswell M., Mottershead J.E. (2013). Finite Element Model Updating in Structural Dynamics.

[13] Grip N., Sabourova N., Tu Y.M. (2017). Sensitivity-based model updating for structural damage identification using total variation regularization. Mech. Syst. Signal Process..

[14] Yan A.M., Kerschen G., De Boe P., Golinval J.C. (2005). Structural damage diagnosis under varying environmental conditions–part I: A linear analysis. Mech. Syst. Signal Process..

[15] Cawley P., Adams R.D. (1979). The location of defects in structures from measurements of natural frequencies. J. Strain Anal. Eng. Des..

[16] Salawu O.S. (1997). Detection of structural damage through changes in frequency: A review. Eng. Struct..

[17] Yang Q.W., Liu J.K. (2009). Structural damage identification by adding given masses. Eng. Mech..

[18] Guan D.Q., Huang Y. (2010). Damage identification of frame structure by means of wavelet analysis of strain mode. Chin. J. Comput. Mech..

[19] Gu P.Y., Deng C., Tang L. (2011). Experimental study on damage identification based on operational strain modal shape. J. Vib. Shock.

[20] Pandey A.K., Biswas M. (1995). Experimental verification of flexibility difference method for locating damage in structure. J. Sound Vib..

[21] Zhang Q.W. (2007). Statistical damage identification for bridges using ambient vibration data. Comput. Struct..

[22] Zong Z.H., Niu J., Wang H. (2012). Research progress of structural Probabilistic Damage Identification Method Based on model validation. China Civ. Eng. J..

[23] Sajid H., Hossam H.A. (2012). A robust method for coupling detection among process variables. Int. J. Process. Syst. Eng..

[24] Widodo A., Yang B.S. (2007). Support vector machine in machine condition monitoring and fault diagnosis. Mech. Syst. Signal Process..

[25] Wang L., Chan T.H.T. Review of vibration-based damage detection and condition assessment of bridge structures using structural health monitoring. Proceedings of the Second Infrastructure Theme Postgraduate Conference: Rethinking Sustainable Development: Planning, Engineering, Design and Managing Urban Infrastructure.

[26] Xu H., Cheng L., Su Z.Q., Guyader J.L. (2011). Identification of structural damage based on locally perturbed dynamic equilibrium with an application to beam component. J. Sound Vib..

[27] Challamel N. (2011). Higher-order shear beam theories and enriched continuum. Mech. Res. Commun..

[28] Yan Y.J., Cheng L., Wu Z.Y., Yam L.H. (2007). Development in vibration-based structural damage detection technique. Mech. Syst. Signal. Process..

[29] Kostrzewski M. (2017). Securing of safety by monitoring of technical parameters in warehouse racks, in high-bay warehouses and high storage warehouses–literature review of the problem. LogForum,.

